# The 3Rs of Animal Research

**DOI:** 10.1016/j.ebiom.2022.103900

**Published:** 2022-02-24

**Authors:** eBioMedicine

On Dec 3, 2021, The Francis Crick Institute issued a press release titled “Gene editing used to create single-sex mouse litters”, describing the ability of scientists to generate all-male or all-female mice with 100% efficiency. At first glance, this might be construed as another example of humans playing God. However, the significance of this technology, as described in Nature Communications, has far-reaching implications relating to the way scientific research and agriculture is conducted. According to James Turner, one of the corresponding authors of the paper, at least 25 000 publications in the past 5 years have used mice in sex-specific research studies. Multiple reasons might explain this preference, such as the study of particular cancers, sex-specific hormones, or reproductive tissues. The farming industry has similar biases, including hens for eggs, cows for milk, and boars for pork, resulting in the culling of a vast number of animals that are considered to be an undesirable sex. These practices are inefficient and ethically questionable, and strategies like this gene editing approach represent a potential means to mitigate such waste.

The use of animals in scientific research is guided by the principles of the 3Rs (replacement, reduction, and refinement), first described in 1959 by WMS Russell and RL Burch in a publication called The Principles of Humane Experimental Technique. The goal of the 3Rs is to find alternatives to animal testing (replacement), to optimise the amount of information obtained from fewer animals (reduction), and to adopt methods that alleviate distress (refinement). These guiding principles aim to improve both the quality of the science and animal welfare, when the use of animals is unavoidable, and has been adopted by many countries who ensure the 3Rs have been fully considered when conducting animal research.

The 3Rs are not a static set of directives and the technological developments that fuel scientific progress also stimulate innovations in the way the 3Rs are accomplished. For example, the development of new cell culture strategies has facilitated the partial replacement of animals in research and *eBiomedicine* has published articles on lab-on-chip technology (eg, a microfluidic device to model lymphatic vessels in head and neck cancer), and the tractable use of organoids to model a variety of tissues (eg, pancreatic cancer and irritable bowel disease). The full replacement of animals has been achieved by improving the in vivo culture methods of primary human cells (eg, a recent review article on culture developments in cystic fibrosis research), maximising the information that can be obtained from patient biopsies (eg, by applying metagenomic Next-generation sequencing to lung biopsies to identify infections and cancer), and through the application of AI and computational methods to model biological processes in silico (eg, to predict immune response in fatal COVID-19).

Reducing the number of animals used in scientific research can be achieved in a variety of ways. For example, using the gene editor CRISPR-Cas9 strategy to produce litters of the desired sex, as mentioned above, is one approach that should (although not yet proven) be translatable into other model organisms. Improvements in experimental design is another tactic and the use of non-invasive procedures, improved sampling techniques, and use of longitudinal study designs could enable more information to be gathered per animal, resulting in fewer animals having to be used overall.

Consideration should also be given to refining methods, such as housing and husbandry protocols (eg, by providing species-specific housing and environmental enrichment) to help animals acclimatise to their surroundings, and by adopting procedural techniques that minimise distress (eg, non-invasive sampling). This is especially important as pain and stress have been proven to alter animal behaviour and physiology, as outlined by the UK's National Centre for the Replacement, Refinement & Reduction of Animals in Research (NC3Rs), resulting in potentially unreliable data.

A small shift in the way preclinical studies are carried out can have a profound impact on animal welfare and the quality of the scientific data obtained. According to the UK Home Office's most recent report, published on July 15, 2021, the Annual Statistics of Scientific Procedures on Living Animals showed a year-on-year decrease in experimental procedures since 2015, with 2020 showing the lowest number of procedures since 2004 (affected in part by the COVID-19 pandemic disrupting research). Although this decline is encouraging, 2•88 million animal procedures were still done in the UK in 2020, and NC3Rs promote several funding schemes to support the adoption of alternative models, tools, and technologies to drive the impact of the 3Rs.

*eBioMedicine* takes animal welfare extremely seriously and we have guidelines in place to ensure that research incorporating animal use has been carried out rigorously. Our editors make sure that primary research articles contain an ethics section that complies with the relevant institutional regulations. Explicit procedures must be provided in the methods section, and the use of analgesics and anaesthesia is scrutinised (the manuscript is rejected if deemed inappropriate). All studies featuring animal procedures require a completed *Animal Research: Reporting of in vivo Experiments* (ARRIVE) checklist prior to publication to maximise the quality and reliability of the published research. We also mandate a Data Sharing Statement to increase data transparency. The preregistration of preclinical trials on publicly available platforms, such as www.preclinicaltrials.eu, is also encouraged to help avoid unplanned duplication of experiments and minimise publication bias in animal studies. We hope that more of our authors will embrace this worthy initiative.

“All models are wrong, but some are useful” is an aphorism attributed to the statistician George Box. At *eBioMedicine*, we are passionate about championing the best preclinical models that yield reliable, relevant, and responsibly conducted translational research. We urge the biomedical research community to help us in this endeavour.

